# Locally-Delivered T-Cell-Derived Cellular Vehicles Efficiently Track and Deliver Adenovirus Delta24-RGD to Infiltrating Glioma

**DOI:** 10.3390/v6083080

**Published:** 2014-08-12

**Authors:** Rutger K. Balvers, Zineb Belcaid, Sanne K. van den Hengel, Jenneke Kloezeman, Jeroen de Vrij, Hiroaki Wakimoto, Rob C. Hoeben, Reno Debets, Sieger Leenstra, Clemens Dirven, Martine L.M. Lamfers

**Affiliations:** 1 Department of Neurosurgery, Brain Tumor Center, Erasmus MC, Dr. Molewaterplein 50, Ee2236, 3015GE, Rotterdam, The Netherlands; E-Mails: r.balvers@erasmusmc.nl (R.K.B.); z.belcaid@erasmusmc.nl (Z.B.); j.kloezeman@erasmusmc.nl (J.K.); j.devrij@erasmusmc.nl (J.V.); s.leenstra@erasmusmc.nl (S.L.); c.dirven@erasmusmc.nl (C.D.); 2 Department of Molecular Cell Biology, Leiden University Medical Center, Leiden, Einthovenweg 20, 2333 ZC, The Netherlands; E-Mails: sanne.vandenhengel@wetsus.nl (S.K.H.); r.c.hoeben@lumc.nl (R.C.H.); 3 Molecular Neurosurgery Laboratory, Brain Tumor Research Center, Massachusetts General Hospital, Boston, MA 02114, USA; E-Mail: HWakimoto@partners.org; 4 Laboratory of Experimental Tumor Immunology, Department Medical Oncology, Erasmus MC Cancer Institute, Rotterdam, 3015 GE, Netherlands; E-Mail: j.debets@erasmusmc.nl

**Keywords:** glioblastoma, oncolytic, cellular vehicles, GSC, T-cell therapy, virotherapy, Delta24-RGD

## Abstract

Oncolytic adenoviral vectors are a promising alternative for the treatment of glioblastoma. Recent publications have demonstrated the advantages of shielding viral particles within cellular vehicles (CVs), which can be targeted towards the tumor microenvironment. Here, we studied T-cells, often having a natural capacity to target tumors, for their feasibility as a CV to deliver the oncolytic adenovirus, Delta24-RGD, to glioblastoma. The Jurkat T-cell line was assessed in co-culture with the glioblastoma stem cell (GSC) line, MGG8, for the optimal transfer conditions of Delta24-RGD *in vitro*. The effect of intraparenchymal and tail vein injections on intratumoral virus distribution and overall survival was addressed in an orthotopic glioma stem cell (GSC)-based xenograft model. Jurkat T-cells were demonstrated to facilitate the amplification and transfer of Delta24-RGD onto GSCs. Delta24-RGD dosing and incubation time were found to influence the migratory ability of T-cells towards GSCs. Injection of Delta24-RGD-loaded T-cells into the brains of GSC-bearing mice led to migration towards the tumor and dispersion of the virus within the tumor core and infiltrative zones. This occurred after injection into the ipsilateral hemisphere, as well as into the non-tumor-bearing hemisphere. We found that T-cell-mediated delivery of Delta24-RGD led to the inhibition of tumor growth compared to non-treated controls, resulting in prolonged survival (*p* = 0.007). Systemic administration of virus-loaded T-cells resulted in intratumoral viral delivery, albeit at low levels. Based on these findings, we conclude that T-cell-based CVs are a feasible approach to local Delta24-RGD delivery in glioblastoma, although efficient systemic targeting requires further improvement.

## 1. Introduction

Glioblastoma is the most frequently diagnosed primary brain tumor in adults, with a median survival of only 15 months after the initial diagnosis [1]. Despite the extensive scientific progress that has been made into the molecular characteristics of glioblastoma, patient prognosis has remained virtually unaltered. As a result, translational research is warranted for the development of new therapeutic agents. Oncolytic virotherapy has been extensively studied for the treatment of glioblastoma and has proven to be a safe and feasible strategy from preclinical [2,3] and clinical safety studies [4,5]. Delta24-RGD is a conditionally replicating oncolytic adenovirus that has demonstrated therapeutic efficacy in preclinical models of glioblastoma [6,7,8]. The Delta24 mutation consists of a 24-base pair deletion in the E1A gene of the serotype 5 adenovirus [9]. This alteration facilitates selective viral replication in cells that harbor altered Rb pathway signaling, which is a common (75%) phenomenon in glioblastoma [10]. The addition of the RGD motif enhances the potency of Delta24 by targeting the virus to alpha (V)-beta(3) integrins that are frequently expressed on tumor cells and vasculature [11]. This omits the dependency on the expression of the Coxsackievirus and Adenovirus receptor (CAR).

One of the major hurdles for virotherapy to target glioblastoma is efficient vector delivery, preferably over a prolonged period of administration [12]. Several strategies have been proposed to overcome this problem [5], of which cellular vehicles (CV) are a particularly interesting option [13]. CV-systems are comprised of cells that are incubated with oncolytic virus *ex vivo*. Subsequently, these cells are administered systemically to employ their intrinsic tropism towards the tumor microenvironment, where efficient delivery takes place in the form of viral transfer or lysis of the CV [14]. Some of the proposed potential advantages of CV are the shielding of viral particles from systemic neutralizing barriers, the ability of dosage amplification by replication of virus within infected vehicle cells and the ability to target tumor cells in multiple administrations.

A myriad of vehicle cells from different tissue lineages have been investigated for their capacity to deliver oncolytic adenovirus into glioblastoma, such as mesenchymal [15,16,17], neural [18,19,20] and adipose-derived stem cells [21]. While other oncolytic viruses have been used in combination with hematological lineage-derived cellular vehicles [22], oncolytic adenovirus has to date not been utilized in such a CV-system. The use of myeloid derived cells as CV for oncolytic adenovirus has been proven effective in prostate cancer xenografts [23]. We have investigated the feasibility of the Jurkat T-cell line as a model for T-cell-based cellular delivery of Delta24-RGD in a glioblastoma stem cell (GSC)-based preclinical xenograft model. The presented study addresses the feasibility of this approach as a step towards targeted delivery of oncolytic adenovirus in combination with an adoptive immunotherapy treatment.

## 2. Methods

### 2.1. Cell Culture

The glioblastoma stem cell line MGG8-Fluc-Mcherry was established and characterized as previously described [24,25]. Tumor spheres were grown in DMEM-F12 supplemented with penicillin-streptomycin, B27, EGF (5µg/mL), FGF(5µg/mL) and heparin(5mg/mL). Tumor spheres were passed by mechanical and chemical dissociation (Life Technologies, Bleiswijk, The Netherlands). For viability assays, cells were grown as monolayers by coating 96-well plates with growth factor-reduced matrigel coating (BD Bioscience, Breda, The Netherlands). The A549 lung adenocarcinoma cell line (ATCC, Manassas, VA, USA) was cultured in DMEM supplemented with 10% FCS and 1% penicillin-streptomycin. Jurkat T-cell E6.1 and Jurkat T-cell-GFP cells were grown in suspension in RPMI supplemented with 10% FCS and 1% penicillin-streptomycin. For systemic delivery, retroviral vectors encoding human CD8α, as well as TCR genes (see below) were used to transduce Jurkat T-cells. The CD8α gene was described previously [26] and the TCRα and β genes originated from the gp100/HLA-A2-specific CTL clone 296 [27]. Following gene transduction, Jurkat T-cells were sorted for TCR expression (as described previously [28]). Our *in vivo* studies, using T-cells expressing a defined TCR, allowed us to use gp100 as a test target antigen for viral treatment of glioma.

### 2.2. Virus Construction and Propagation

Delta24-RGD was constructed as previously described [9]. For the construction of Delta24-RGD-GFP, a set of previously developed plasmids was used to create the virus HAdV-5.Δ24.Fib.RGD.eGFP. This virus combines the unique properties of Delta24-RGD with a replication-dependent expression of the eGFP imaging marker, as a result of incorporating eGFP in the viral promoter-driven E3 region [29]. To this end, the RGD motif was excised from the plasmid, pVK526 [30], by NdeI + PacI digestion and re-ligated into the plasmid, pShuttle-ΔE3-ADP-EGFP-F2 [29], resulting in pShuttle-ΔE3-Fib.RGD.ADP-EGFP. After removal of the kanamycin resistance gene (by ClaI digestion and re-ligation), PacI + AatII digestion was used to isolate the fragment containing the ΔE3-Fib.RGD.ADP-EGFP sequence, which was recombined with SpeI-linearized pAdEasy-1 [30], resulting in pAdEasy-ΔE3-Fib.RGD.ADP-EGFP. The 24-bp deletion was introduced in the plasmid, pSh + pIX [31], by replacement of the SspI-to-XbaI fragment with the corresponding fragment from the plasmid pXE.Δ24 [32], resulting in the plasmid, pSh + pIX.Δ24. The full-genomic sequence of HAdV-5.Δ24.Fib.RGD.eGFP was constructed by recombination in *E. coli* of pAdEasy-ΔE3-Fib.RGD.ADP-EGFP with pSh + pIX.Δ24. The virus was rescued in 911 cells [33], using a previously described protocol. [30] To prevent heterologous recombination with the viral E1 sequence present in the 911 genome, upscaling of the virus was performed in A549 cells. After preparation of the virus stock, the presence of Δ24 and Fib.RGD was confirmed by PCR and restriction analysis.

### 2.3. Delta24-RGD Infection and Replication Assay

Jurkat T-cells were infected with Delta24-RGD at multiplicities of infection (MOI) 1, 10, 50, 100, 500 and 1,000 by plating cells for 2 h in serum free RPMI at room temperature. After 2 h, cells were washed and spun down twice in serum supplemented RPMI. Subsequently, cells were plated in triplicates of 1 × 10^3^ cells per well in flat-bottomed 96-well plates. Cells were allowed to proliferate for 4 and 6 days, after which we performed the Cell Titer GLO viability assay (Promega, Leiden, The Netherlands), as described by the manufacturer. For the treatment of MGG8-spheres, the MOI was calculated based on the seeded cells counted from dissociated spheres. Cells were incubated for one day in which spheres form through adherence, and incubation followed 24 h post-seeding, making the MOI in our hands reproducible and accurate.

Transfer of Delta24-RGD-GFP from Jurkat T-cells towards MGG8-Mcherry-FLuc was assessed by infecting Jurkat T-cells at MOI 0, 1, 10 for 24 h, washed twice and co-cultured at a 1:1 ratio with MGG8 cells for 5 days. Microscopic examination and image capture were performed on a conventional wide-field fluorescence microscope. For these experiments, MGG8 cells were cultured on growth factor-reduced matrigel coating.

The replication assay was performed with the above-described infection protocol at MOI 10, 50 and 100. Jurkat T-cells were harvested 1.5 h and 4 days post-infection. Pellets and supernatants were collected and separately freeze-thawed three times, and subsequently, pellets were reconstituted in medium to equal volumes, as present in the supernatants. After 48 h, A549 cells were fixed with ice-cold methanol, and the Ad Rapid Titer plaque-forming assay (Clontech, Saint-Germain-en-Laye, France) was performed according to manufacturer's protocol. Experiments were performed twice, in triplicates.

### 2.4. T-Cell Migration Assays

Suspensions of 1 × 10^6^ cells/ml Jurkat T-cells in RMPI were prepared. Cells were infected with Delta24-RGD dilutions at an MOI of 10, 50 and 100 in 1 mL of serum free RPMI. Cells were incubated for 2 h and subsequently washed twice with serum supplemented RPMI and incubated for another 2, 24 or 48 h. Hereafter, 5 × 10^5^ cells were inserted onto matrigel-coated transwell chamber inserts with 5-μm pores (Corning Inc., Amsterdam, The Netherlands). Cells were allowed to migrate into the bottom compartment for 12 h, after which cell numbers were quantified by performing a Cell Titer GLO viability assay (Promega, Leiden, The Netherlands) according to the manufacturer’s instructions. Experiments were performed twice, in duplicates.

### 2.5. *In Vivo* Experiments

All animal experiments were performed in accordance with the local Animal Ethical Committee, Erasmus MC Rotterdam. Experiments were performed on 10–12-week-old NOD-SCID (Non-obese diabetic severe combined immunodeficient) female mice (strain C.B-17/IcrHantmhsd-Prkdcs), Harlan, (Horst, The Netherlands). On Day 0, intra-striatal injections of 5 × 10^4^ MGG8-Fluc-Mcherry cells were performed as described previously [34]. In short, a burr hole was drilled 2.5 mm lateral and 0.5 mm anterior to the bregma, through which 5 × 10^4^ MGG8-Fluc-Mcherry cells in 5 µL PBS were injected at a depth of 3 mm. After 14 days, mice were treated with intratumoral injections through the previously established burr hole. Treatment consisted of 5 µL PBS, Delta24-RGD 5 × 10^6^ IUs in 5 µL PBS or 5 × 10^4^ Jurkat T-cells infected with MOI 100 Delta24-RGD in 5 µL PBS. Jurkat T-cells were incubated in Delta24-RGD (MOI-100) 2 h prior to intratumoral injections. Mice were sacrificed upon symptoms of tumor burden or when more than 20% weight loss occurred.

For systemic delivery of Jurkat T-cells infected with Delta24-RGD, mice were grafted with MGG8-Fluc-Mcherry, as described above. After 14 days, treatment consisted of tail vein injections with 5 × 10^5^ Jurkat T-cell-gp100^TCR^ cells pretreated with Delta24-RGD MOI 100 for 2 h, as described above. Animals were treated with one (*n* = 3), two (*n* = 3) or three (*n* = 3) subsequent injections with a two-day interval. Animals were sacrificed after 48 h, 96 h and 144 h after the first injection, allowing a cumulative dosing effect in the latter two time points. Control animals were treated with equivalent dosages of Delta24-RGD through tail vein injections. Brains were snap frozen and assessed for viral delivery by hexon staining.

### 2.6. Immunohistochemistry

Snap frozen tumor tissue sections (8–10 μm) were cut on a cryotome. Sections were fixed with acetone. After permeabilization (Triton-X 0.01%) and blocking, slices were incubated with goat anti-hexon (Millipore, Billerica MA, USA) and mouse anti-vimentin (from DAKO, Heverlee, Belgium). Negative controls were performed by omitting the primary antibodies. Counterstaining was applied using Vectastain mounting medium, including DAPI, according to the manufacturer’s instruction (Vector Labs, Peterborough, U.K.).

### 2.7. Statistics

Statistical analyses were conducted and illustrated in SPSS Statistics Software 19 (IBM, Amsterdam, The Netherlands).

## 3. Results

### 3.1. Delta24-RGD Efficiently Infects and Replicates in GSC and Jurkat T-Cells

**Figure 1:**
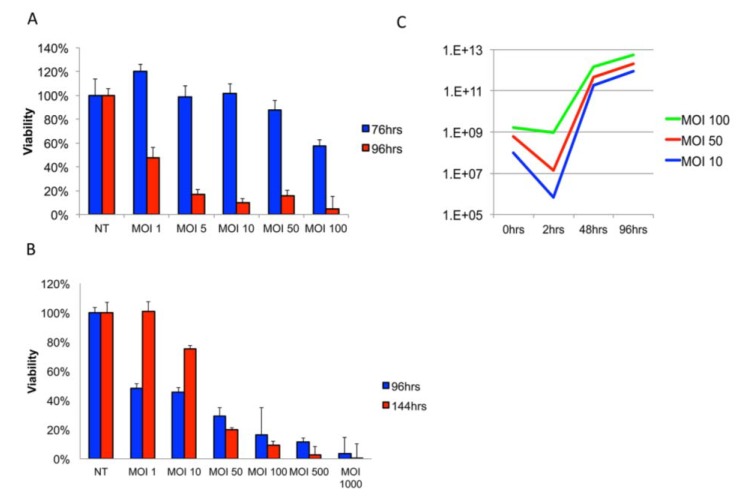
Delta24-RGD effectively infects, replicates and amplifies in Jurkat T-cells. (**A**) Viability assay on MGG8-GSC neurospheres infected with a dose range of Delta24-RGD; (**B**) viability assay on Jurkat T-cells infected with a dose range of Delta24-RGD. Note the increased viability at MOI 1–10 after 144 h (**C**). The viral titer assay for Delta24-RGD-treated Jurkat T-cells after two wash steps at indicated time points. Lines are representative of viral yield at indicated time points post-infection. (IUs = viral infectious units).

Delivery of Delta24-RGD by Jurkat T-cell-CV was assessed in a series of *in vitro* assays. First, the optimal dosage for Delta24-RGD-mediated lysis of MGG8 GSCs was assessed by viability assay (Figure 1A). MGG8 tumor neurospheres were found to be susceptible to Delta24-RGD-induced oncolysis with an IC_50_ value of MOI 0.89 at 96 h post-infection (R^2^ = 0.92–0.99).

Efficient adenoviral infection of leukocytes has been described as problematic in several reports [35,36], while others have demonstrated efficient use of conditionally replicating adenoviruses in a subset of T-cell derived neoplastic cell lines, including Jurkat T-cells [37,38]. To investigate the infectivity and replication capacity of Delta24-RGD in Jurkat T-cells, we performed viability and viral titer assays on cells and supernatants. A dose-response effect of Delta24-RGD on Jurkat T-cell viability at 96 h post-infection was found (Figure 1B). However, after 144 h, cells exposed to MOI 10 or lower had repopulated the well. This is reflected in the IC_50_ values of MOI 9.2 at 96 h and MOI 20.4, at 144 h (R^2^ 0.92–0.97). At MOI 50 and higher, the *in vitro* administration of Delta24-RGD was sufficient to warrant efficient lysis and prevent population renewal.

The ability of Delta24-RGD to replicate and produce progeny in Jurkat T-cells is demonstrated in Figure 1C. The infection of Delta24-RGD in Jurkat T-cells was dose-dependent and reached a saturation level at MOI 100. This was observed by a decrease in viral concentration in supernatants at 2 h post-infection at MOI 10 and 50 of 99.99% and 98% of input virus, respectively, whereas only a 7% decrease in input virus was found at MOI 100 (Figure 1C, blue bars). In retrospect, we also derived an explanation from this data for the ability of Jurkat cells to repopulate after infection with an MOI below 10, since the infectivity will not suffice to cause timely replication in Jurkat cells. A significant amplification of input viral load was demonstrated at all three dosages by 48 h (5.28 × 10^2^–1.82 × 10^3^-fold), which further increased at 96 h (2.12 × 10^3^–9.16 × 10^3^-fold). Interestingly, the relative amplification was highest in MOI 10-treated cells, suggesting a plateau for the replication-to-infection ratio.

### 3.2. T-Cells Efficiently Deliver Delta24-RGD to GSCs *In Vitro*

**Figure 2:**
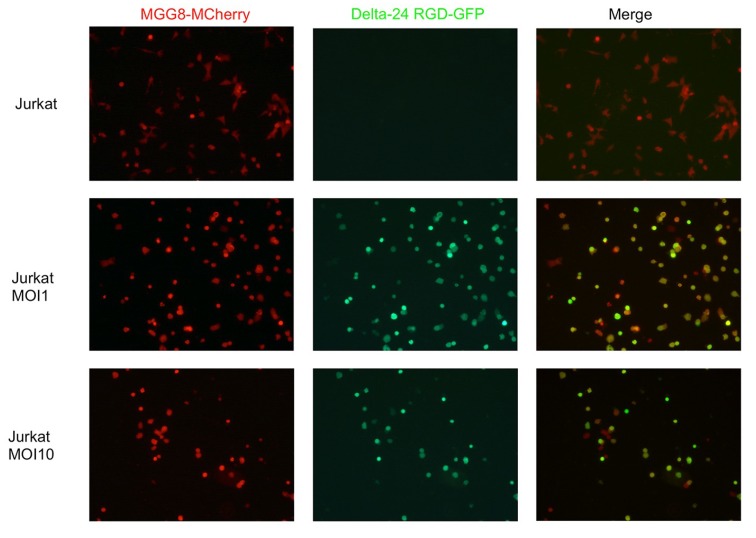
Jurkat T-cells efficiently deliver Delta24-RGD onto MGG8-GSCs *in vitro*. Cells were co-cultured for 96 h after prior incubation (24 h) of Jurkat T-cells with Delta24-RGD at the indicated dosages. (**Left**) mCherry fluorescence as assessed by conventional fluorescence microscopy with 25× optic zoom. (**Middle**) GFP fluorescence, indicating the presence of Delta24-RGD-GFP within cells. (**Right**) A merge of both mCherry and GFP results, demonstrating colocalization (yellow cells) indicative of Delta24-RGD-GFP transfer onto MGG8-mCherry cells.

Since both Jurkat T-cells and MGG8 cells were found to be susceptible to Delta24-RGD-mediated oncolysis, the ability of Jurkat T-cells to function as a carrier cell to deliver virus was tested. To this end, we utilized a GFP-expressing variant of Delta24-RGD with a GFP-imaging cassette inserted into the E3 region of the viral genome. First, we tested the ability of (twice washed) Jurkat T-cell-CV to deliver virus onto MGG8 cells in a co-culture experiment. Delta24-RGD-incubated Jurkat T-cells were able to efficiently transfer virus onto MGG8 cells, as demonstrated by the accumulation of hexon-positive Jurkat T-cells and MGG8 cells over a time-span of 96 h post-infection in co-cultures (Figure 2). Similar to viability assays with Delta24-RGD monotherapy, survival of both Jurkat T-cells and MGG8 was dose dependent in this co-culture experiment, as demonstrated by a greater loss of both cell types at MOI 10, as compared to MOI 1.

### 3.3. The Migratory Capacity of T-Cells Is Preserved Following Short Incubation Times with Delta24-RGD

**Figure 3:**
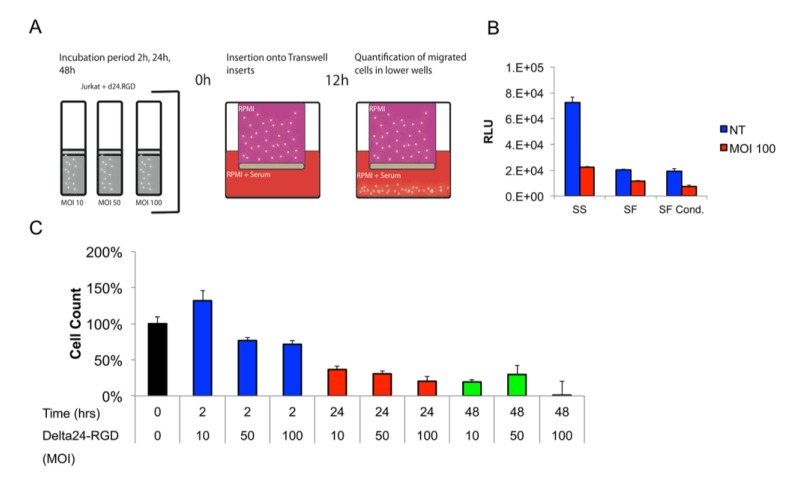
The Jurkat T-cell cell migration ability is influenced by Delta24-RGD dosage and incubation time. (**A**) Schematic illustration of the transwell experiments; (**B**) titration of Delta24-RGD dosage and incubation window. All time points and dosages are significantly different (*p* < 0.05) when compared to the non-treated (NT) control. Note the increased cell count in MOI 10 after 2 h, which could be indicative of T-cell activation. (**C**) Quantification of Jurkat T-cell tropism towards serum-supplemented (SS), serum-free (SF) and MGG8-conditioned serum-free medium (SF cond.). All comparisons between NT and Delta24-RGD resulted in *p* < 0.05. SS NT *vs*. SF/SF cond. NT were both *p* < 0.05.

The brain tumor tropism of stem cells and leukocytes is influenced by secreted cytokines and chemokines by tumor and stromal cells [39,40,41]. Furthermore, the components of the extracellular matrix proteins can both attenuate and enhance T-cell infiltration into brain parenchyma [42]. Little is known about the role of adenoviral infection on leukocyte migration towards tumors. However, a positive influence on migration with low-dosages of oncolytic VSV (vesicular stomatitis virus) has been reported by others [43,44]. To address the influence of both Delta24-RGD and the aforementioned factors on Jurkat T-cell migration was assessed in a transwell migration assay (schematic representation in Figure 3A). To be able to fine-tune future administration of cells *in vivo*, separate time-points were chosen to correct for the latency between infection and administration. Both commercial extracellular matrix protein, as well as MGG8-derived matrix coating slightly reduced (non-infected) Jurkat T-cell migration towards the lower compartment (data not shown). The effect of Delta24-RGD infection on Jurkat T-cell tropism toward serum-supplemented medium was assessed in ECM-coated transwell filters at 2, 24 and 48 h post-infection (MOI 10, 50 and 100). A short virus incubation time of 2 h best conserved the migratory capacity of the Jurkat T-cells when compared to non-infected controls. Interestingly, dosing at MOI 10 led to increased migration, which may be a result of virus-mediated activation of the Jurkat T-cells [42]. Prolonged incubation (24–48 h) had a significant inhibitory effect on Jurkat T-cell migration (Figure 3B).

Furthermore, Jurkat T-cells migrated most efficiently to serum-rich medium. Both serum-free (EGF and bFGF supplemented) as well as MGG8-conditioned medium led to similar levels of reduction of Jurkat T-cell migration over matrix-coated inlays (Figure 3C).

### 3.4. Delta24-RGD-Loaded T-Cells Demonstrate Intraparenchymal Tumor Tropism and *In Vivo* Viral Transfer

**Figure 4:**
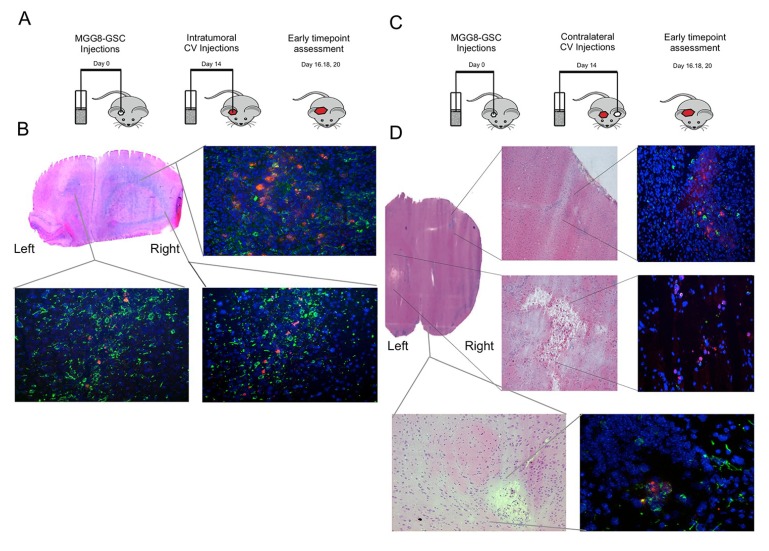
Intratumoral and contralateral Jurkat T-cell injections target GSC *in vivo*: (**A**) Schematic representation of the injection site and early time point assessment of cellular vehicle (CV)-Delta24-RGD distribution experiments. (**B**) Coronal HE (hematoxylin and eosin stain) section of a mouse brain (Day 4 post-injection) with MGG8-derived tumor in the right hemisphere (injection site) and spreading to the left hemisphere. Inlays demonstrate tumor and Delta24-RGD distribution at three localizations after intratumoral virus injection. (**Upper right**) Tumor core (vimentin = green, adenoviral hexon protein = red); (**lower left**) contralateral hemisphere; (**lower right**) right hemisphere basal localization of tumor and Delta24-RGD. (**C**) Schematic representation of the injection sites and early time point assessment after CV-Delta24-RGD injections into the contralateral hemisphere. (**D**) Coronal HE section with inlays demonstrating in the **upper left** and **right panel** the cortical invasion of localized tumor cells in both HE and immunofluorescence images. The **upper right** inlay demonstrates hexon distribution in the upper right hemisphere (contralateral from Jurkat T-cell-CV injection) adjacent to dispersed invasive MGG8 cells. The **lower left** and **lower right** panel illustrate the necrotic cavity resulting from the contralateral injections of Jurkat T-cell-CV, together with fluorescence images demonstrating hexon-positive cells locally. In the **bottom** two inlays, a second localization of Delta24-RGD localized in the proximity of tumor cells is demonstrated from the basolateral margins of the right hemisphere.

Based on the optimal dosing and incubation time *in vitro*, we proceeded to assess the *in vivo* potential of CV-based delivery of Delta24-RGD. For this, virus-loaded Jurkat T-cell CVs were injected intratumorally in MGG8-bearing mice (schematic representation of the experiment is provided in Figure 4A). Jurkat T-cells were incubated for 2 h at MOI 100 to achieve maximal infection and minimal loss of migratory capacity. First, the distribution of viral hexon-positive cells was assessed at early time points (24–144 h) post-intratumoral Jurkat T-cell delivery. Hexon staining revealed the presence of adenovirus, both in the core of the tumor, as well as at the peripheral margins (such as the contralateral hemisphere) of the tumor, where infiltrating single tumor cells reside (Figure 4B). Jurkat T-cell-GFP cells were especially present in (and around) the tumor core, suggesting preferential targeting for the xenografted tumor cells (Figure 4C). Encouraged by these results, the distribution of virus after contralateral injections (in the non-tumor-bearing hemisphere) at early time-points was assessed (schematic representation in Figure 4D). As expected, hexon staining was demonstrated within the Jurkat T-cell injection site, suggestive of the T-cell-based Delta24-RGD distribution, which left a necrotic cavity (Figure 4E). Strikingly, additional hexon staining was detected in the tumor-bearing hemisphere in small tumor colonies at the invading margins of the tumor. These results demonstrate the intraparenchymal tropism of CV to the tumor, subsequently resulting in the *in vivo* transfer of oncolytic adenovirus to distant tumor cells (Figure 4E).

### 3.5. Intratumoral Delivery of Delta24-RGD by T-Cells Leads to Prolonged Survival

**Figure 5:**
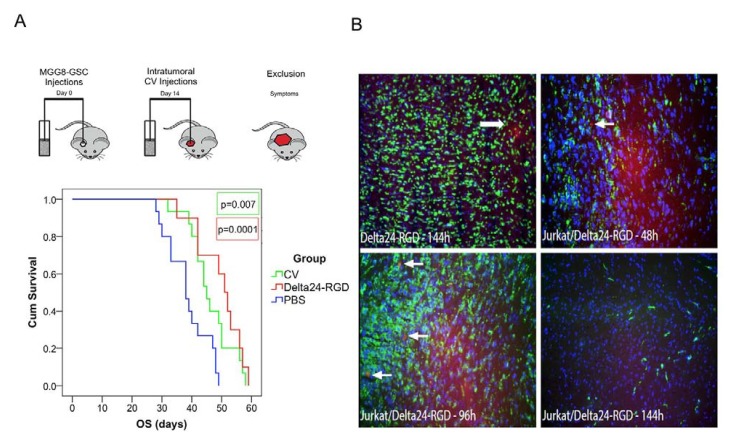
Intratumoral injection of Jurkat T-cell-CV leads to prolonged survival, and systemic therapy is a feasible mode of administration for intracranial targeting of MGG8 xenografts. (**A**) Schematic representation of the survival experiment with PBS and Delta24-RGD monotherapy as control groups for the Jurkat T-cell-CV treatment of MGG8 xenografts. Kaplan Meier graphs demonstrating a significant treatment effect of both intratumoral Delta24-RGD, as well as Jurkat T-cell-CV treatment. *p*-values are derived from a Wilcoxon log rank pairwise comparison to PBS-treated controls. There was no significant difference between CV and Delta24-RGD monotherapy. (**B**) MGG8 xenografts attract systemically-administered Delta24-RGD-loaded Jurkat T-cell-gp100^TCR^. (**Upper left**) Intratumoral controls after systemic Delta24-RGD monotherapy with localized hexon staining (arrow). (**Upper right**) Intratumoral hexon-positive cells (arrow) 48 h after Jurkat T-cell delivery, indicative of the homing of Jurkat T-cells to the tumor. (**Bottom left**) Similar hexon-positive cells (arrows) at 96 h post-treatment. (**bottom right**) No hexon-positive cells were noted at 144 h post-treatment.(vimentin = green, adenoviral hexon = red).

Therapeutic effect of Jurkat T-cell-mediated Delta24-RGD delivery was compared to both PBS and Delta24-RGD intratumoral injections. Delta24-RGD-loaded T-cells (CV) prolonged the overall survival of mice bearing MGG8 orthotopic xenografts to a similar extent as direct intratumoral injection of Delta24-RGD. When compared to PBS-treated controls (mean: 38.6 days), both treatment conditions prolonged survival significantly (CV mean survival: 46.2 days, Log Rank *p* = 0.007; and Delta24-RGD mean: 49.6 days, Log Rank *p* < 0.001) (Figure 5A). There was no difference in survival between the CV and Delta24-RGD treatment groups (*p* = 0.251).

### 3.6. Delta24-RGD-Loaded T-Cells Demonstrate Tropism for Intracranial Tumors

Based on these results, a study was undertaken to assess the ability of CV to deliver Delta24-RGD after systemic tail-vein injections. To this end, MGG8-bearing mice received i.v. injections of Delta24-RGD-loaded Jurkat T-cells. The control group received i.v. injections of similar doses Delta24-RGD virus. To enhance CV tropism towards MGG8, Jurkat T-cells were transduced with a T-cell receptor that specifically recognizes gp100 antigen presented bij HLA-A2. Gp100 is a tumor-antigen commonly expressed in Glioblastoma and MGG8 *in vitro* and *in vivo* (data not shown) [45,46]. Intratumoral dispersion of CV and/or infection of the tumor cells was assessed by hexon staining after sacrificing the animals after 48–144 h post-treatment. Analyses of the brains revealed that direct intravenous injection of Delta24-RGD did not lead to infection of MGG8 tumors in any of the analyzed animals, with the exception of a small vascular structure in one animal at 144 h post-injection (Figure 5B, upper left). Contrary to virus-only injections, in mice that received Delta24-RGD-loaded Jurkat T-cells, we observed multiple small hexon-positive cells at 48 and 96 h post-injection (Figure 5B, upper right and lower left). At 144 h after systemic administration, however, no hexon-positive cells were detected, nor where there any indications of intratumoral viral spreading observed (Figure 5B, lower right).

## 4. Discussion

In the current study we investigated the feasibility of T-cell-mediated delivery of Delta24-RGD to glioblastoma. The application of cellular vehicles to deliver oncolytic viruses potentially overcomes several limitations of oncolytic virotherapy as a therapeutic alternative for glioblastoma (e.g., limited window of drug administration). For this reason, the use of leukocytes as cellular vehicles would be a step up in studies investigating targeted autologous T-cells, as a means to combine oncolytic virotherapy with immunotherapy. The data presented here demonstrate the ability of Delta24-RGD to efficiently infect and amplify viral progeny in a T-cell-derived cellular vehicle system. Indeed, T-cell cells were found to succumb to Delta24-RGD infection, albeit at higher MOIs compared to the dose ranges used in glioma cell cultures [6]. This is congruent with a previous study by Yotnda *et al.* [38], demonstrating that higher dosages are needed to efficiently start replication and lysis in leukocytes. This can be accomplished by inserting the RGD-motif into the surface exposed loop of the fiber-knob, which results in increased infectivity.

Based on these *in vitro* findings, we set-up a proof of concept study, which demonstrated that intratumoral CV-based delivery of Delta24-RGD yields comparable results to direct Delta24-RGD injections with regard to therapeutic efficacy. Importantly, delivery of Delta24-RGD into peripheral tumor regions was demonstrated after injections of cellular vehicles into the contralateral hemisphere in GSC xenografts. This indicates the strong tropism and migratory capacity of virus-loaded T-cells in the tracking of tumor cells in the brain environment. Moreover, systemically delivered Delta24-RGD-loaded CVs were detected in small numbers within the orthotopic xenografts within 48–96 h, although efficient virus handover to the tumor was not observed in this setting. We conclude that T-cells have the ability to serve as cellular vehicles to deliver Delta24-RGD to glioblastoma at a distance, in particular to infiltrating tumor cells in the brain parenchyma. This may suggest a potential for CV approaches as a post-surgical adjuvant therapy, where CVs may be deposited in the resection cavity to track residual and surgically-inaccessible tumor cells.

Several hurdles for successful implementation of this T-cell-mediated CV delivery strategy have been addressed. Our *in vitro* studies demonstrate an inhibitory effect of Delta24-RGD infection on Jurkat T-cell migration in tropism assays, which is dependent on both incubation time, as well as viral dosage. Intriguingly, lower MOIs seemed to increase tropism in our transwell assay. Similar enhancement of the T-cell migratory phenotype was reported in a VSV-based cellular vehicle system [44]. It remains to be determined whether infection with lower MOIs would further improve efficient delivery of Delta24-RGD *in vivo*. At present, lower dosages could not be applied in our xenograft model for the risk of developing hematological malignancy from remaining Jurkat T-cells that escape from viral infection; however, the use of an autologous T-cell population may possibly circumvent this problem. As shown, tail vein injections in orthotopic xenograft-bearing mice did not lead to widespread intratumoral delivery of Delta24-RGD. The administration of L1210 leukemic cells in a syngeneic CV model did yield appreciable intracerebral carrier cell infiltration in a VSV delivery model for lung cancer [22], indicating that the choice of oncolytic virus or tumor type may influence intracranial tropism. With regard to this route of administration, it is important to consider that administration through injections into the common carotid artery may increase intratumoral delivery of CVs to the brain, as demonstrated by others for Delta24-RGD delivery by mesenchymal stem cells [15].

The *in vitro* transwell migration assays demonstrated that MGG8-conditioned medium was less effective for the attraction of T-cells compared to serum-supplemented medium. This could either be due to immunologically repressive factors secreted by the tumor or the relative abundance of chemo-attractants available in serum-supplemented medium for T-cells. Recent studies have shown that the secretion of several chemokines plays a crucial role in the influx of both mesenchymal stem cells, as well as lymphocytes in the glioblastoma microenvironment [39,47,48]. Further investigation is warranted to appreciate which factors contribute to CV tropism, and the development of patient-tailored vectors (*i.e.*, specific TCRs, chemokines, suicide genes) may need to be included into the CV system to enhance therapeutic efficacy.

We conclude that CV-mediated delivery of Delta24-RGD holds promise for future therapeutic application. The current results warrant further investigations into the delivery of Delta24-RGD by T-cells, possibly in combination with adoptive immunotherapy strategies.
